# Attitudes and Knowledge of Australian Gastroenterologists Around the Use of Medicinal Cannabis for Inflammatory Bowel Disease

**DOI:** 10.1093/crocol/otaa045

**Published:** 2020-06-08

**Authors:** Melissa J Benson, Sarah V Abelev, Crispin J Corte, Susan J Connor, Iain S McGregor

**Affiliations:** 1 Lambert Initiative for Cannabinoid Therapeutics, The University of Sydney, Sydney, NSW, Australia; 2 Faculty of Science, School of Psychology, The University of Sydney, Sydney, NSW, Australia; 3 Brain and Mind Centre, The University of Sydney, Sydney, NSW, Australia; 4 Department of Gastroenterology, Royal Prince Alfred Hospital, Sydney, NSW, Australia; 5 Faculty of Medicine and Health, Central Clinical School, The University of Sydney, NSW, Australia; 6 Department of Gastroenterology, Liverpool Hospital, Sydney, NSW, Australia; 7 Ingham Institute of Applied Medical Research, Sydney, NSW, Australia; 8 The University of New South Wales, Sydney, NSW, Australia

**Keywords:** medicinal cannabis, gastroenterology, surveys and questionnaires, inflammatory bowel diseases

## Abstract

**Background:**

Medicinal cannabis (MC) is being used for symptomatic relief by many patients with inflammatory bowel disease (IBD), often independently of clinical guidance. Such use presents challenges for supporting clinicians. The aim of this study was to determine the current attitudes, knowledge, and experience of gastroenterologists toward patient use of MC for symptom management in IBD.

**Methods:**

Australian gastroenterologists (*n* = 70) and trainees (*n* = 23) completed an anonymous, 30-item questionnaire, probing their knowledge, attitudes, and experience with MC in managing IBD. Survey data were collected between April and August 2019.

**Results:**

Thirty-nine percent of survey respondents reported having patients using MC; however, only a minority supported use of MC in IBD (21%) or expressed a desire to prescribe (28%). Only 6% claimed good understanding of current patient access pathways and only 31% felt comfortable discussing MC with their patients. Some respondents (20%) cited adverse side effects as a reason for not wanting to prescribe, with driving impairment (64%) and impacts on the developing brain (56%) cited as significant concerns. Nonetheless, MC was ranked as less hazardous than corticosteroids, immunomodulators, and biologics by most respondents, and many (53%) were encouraging of patient participation in future clinical trials.

**Conclusions:**

Specialist support for the use of MC in IBD patients is relatively low, potentially reflecting the lack of experience and knowledge with MC, uncertain evidence for efficacy, and the often-unorthodox nature of current MC use in patients. This situation may change rapidly with increased familiarity, evidence development, and education around MC prescribing.

## INTRODUCTION

The chronic nature of inflammatory bowel disease (IBD), alongside the low rates of remission achieved with established therapies have led many patients to trial alternatives to conventional treatments, including the increasing use of medicinal cannabis (MC) products. A recent survey of Australians (*n* = 1388) who were self-medicating with cannabis products found that 12.4% were using cannabis to manage gastrointestinal conditions (including IBD).^1^ A recently completed parallel survey of Australian IBD patients (*n* = 838) found that 25% were either currently using or had previously used cannabis to manage their condition.^2^

Australia has one of the highest incidence rates of IBD globally and IBD is recognized as a growing health problem in the community.^3, 4^ The annual incidence rate of IBD in Australia is 29.6 per 100,000 person-years with a prevalence of around 0.2%–0.3%.^4, 5^ Furthermore, an estimated 30%–35% of IBD patients are refractory/poorly managed with existing best-practice therapeutic options,^6^ indicating an urgent need for new and alternative therapies.

Legal access to MC was established in Australia in late 2016 and is regulated by the Therapeutic Goods Administration (TGA), a Federal government organization analogous to the US Food and Drug Administration and the European Medicines Agency. Australian regulations allow for prescription of regulated, pharmaceutical-grade cannabis products through two main schemes that govern access to unregistered medicines; the Authorized Prescriber scheme, where a clinician gains approval to prescribe a specific MC product for a specific class of patients; and the Special Access Scheme B, where a clinician seeks TGA (and state) approval on a case-by-case basis.

At the time of writing >18,500 Australian patients have gained official approval to use MC products for a range of refractory conditions.^7^ However, approvals for use of MC specifically for IBD are very low (only 51 Special Access Scheme B approvals at the time of writing)^8, 9^ and our recent survey shows that IBD patients predominantly use illicit cannabis products rather than officially sourced MC for treatment.^2^ Legal products include mostly oral formulations (ie, oils, sprays, and capsules) containing cannabidiol (CBD) and/or ∆9-tetrahydrocannabinol (THC) in various concentrations and ratios, whereas illicitly sourced products typically involve THC-dominant cannabis plant material that is smoked in joints or bongs.^2^

Robust unequivocal evidence to support the use of MC for management of IBD is currently lacking although this is clearly not deterring many patients from self-medicating with cannabis and reporting improvements (typically without clinician supervision or guidance).^2^ This is an important emerging issue within the gastroenterology community^10^; despite mixed evidence of efficacy, IBD patients continue to self-medicate at increasing rates and clinicians will need to address such use in patients, whether they support MC use or not.

At a mechanistic level there is a rationale for MC use in IBD. The endocannabinoid system strongly influences GI function^11^ and cannabinoids promote anti-inflammatory and wound healing processes in preclinical models of IBD.^12–14^ Published surveys of IBD patients describe self-reported improvements in pain, diarrhea, and poor appetite with MC.^10–14^ Disease severity appears to drive MC use^15–19^ with cannabis-using IBD patients reporting more problematic histories involving greater pain, poorer quality of life, and a history of surgical interventions. Clinical trials of MC in IBD are few in number and generally low in quality. Results suggest reduced steroid dependency and improved appetite and sleep in Crohn’s disease^20^ and some clinical improvements in ulcerative colitis,^21, 22^ but without clear effects on resolution of inflammation. Recent Cochrane reviews concluded that the effects of cannabis on Crohn’s disease^23^ and ulcerative colitis^24^ are uncertain and that no conclusions can be drawn from the existing evidence base to support MC as a treatment for these diseases. Further clinical research is clearly warranted with both subtypes of IBD.

The use of MC by IBD patients poses a challenge to their treating physicians who must balance their patients’ use of unconventional and often unregulated cannabis products with optimized conventional treatment provision. Currently, the attitudes, knowledge, and experience of Australian gastroenterologists regarding the use of MC in IBD are unknown. We therefore conducted an anonymous survey to ask trainees and specialists about their clinical experience with MC, perceived knowledge of efficacy, safety and perspectives on MC regulation. This survey was run in parallel with another examining IBD patient perspectives and experiences with MC in Australia^2^ with the aim of gaining complementary patient and clinician perspectives on the current landscape.

## METHODS

### Study Design

We administered a cross-sectional anonymous survey in both online and paper format to qualified gastroenterologists, and to doctors in their final 3 years of gastroenterology advanced training in Australia. This was achieved via mail out conducted by IQVIA using their database of Australian gastroenterologists (*n* = 1078), or via an online survey hosted by Research Electronic Data Capture (REDCap) through the University of Sydney. Paper surveys were also administered at educational meetings by study investigators. The online survey was promoted as an option on the paper survey and was advertised through University of Sydney websites. Responses were collected between 17 April and 8 August 2019. Eligible respondents were currently registered medical practitioners in Australia with gastroenterology training and willing to provide consent. There was no requirement for respondents to have had experience with MC in their IBD patient population. Respondents were required to confirm that they had only completed the survey once, either in paper or online format. The survey took 5–10 min to complete. There was no reimbursement or financial incentive for taking part in the voluntary survey. A copy of the survey is provided in Supplementary Material.

The term “medicinal cannabis” used in this study refers to the term understood by lay people. That being, any legal or illegal cannabis-based product used for the primary purpose of treatment or symptom alleviation of a self-identified health condition. This does not imply that the cannabis product was indicated or prescribed by a health professional. There are more than 100 MC products available in Australia through TGA schemes for unregistered medicines, involving an array of different formulations (oils, capsules, sprays, and plant material) with varying cannabinoid profiles.

### Questionnaire

A 30-item questionnaire (see Supplementary Material) was developed based on prior surveys assessing knowledge of MC in other Australian clinical specialties.^25, 26^ Specifically, the survey structure was based upon a recent design administered to general practitioners (GPs)^26^ with questions amended to be specific to IBD clinicians. The survey was reviewed by gastroenterologists to ensure appropriate questions were asked. The survey included items to interrogate:

a) Demographics including profession, age, gender, years in gastrointestinal speciality, average hours spent in clinical practise per week, and geographical area serviced.
b) Current IBD patient cohort and patient satisfaction.
c) Experiences with alternative and complementary therapies in IBD patients.
d) Experiences with cannabis in IBD patients.
e) Perspectives on how cannabis should be accessed and used for IBD.
f) Attitudes toward MC regulation in Australia.

A space for open-ended comments was offered at the end of the survey. Respondents were asked if they were interested in being involved in future clinical trials of MC in IBD. If agreed, they were provided a nonlinked platform to provide their contact details (online) or a separate contact form (paper)—neither of which could be linked to survey responses to ensure anonymity.

## ETHICAL CONSIDERATIONS

The survey was approved by the University of Sydney Human Research Ethics Committee (Ref: 2019/116) and carried out according to the National Statement on Ethical Conduct in Human Research (2007). All participants were required to acknowledge they had read the linked Participant Information Statement and confirm consent to the study with a checkbox prior to initiating the survey online or on paper.

## RESULTS

### Study Population

A total of 99 respondents initiated a survey response. Six respondents were excluded from the final analysis as they did not confirm to which profession they belonged (ie, Gastroenterologist; in training). The final data set comprised 93 respondents. Most responses were completed in paper format (*n* = 83), fewer electronically (*n* = 10), with 98% of respondents completing all survey items.

Demographic and practice details of the survey population are shown in Table 1. The majority of respondents were male gastroenterologists practicing for greater than 10 years, mostly in a metropolitan, public hospital setting. The survey respondent population was demographically representative of the broader Australian gastroenterology cohort (based on comparisons to the most recent National Health Workforce Dataset^27^; Table 1).

**Table 1.** Demographic and clinical practise characteristics of survey respondents compared to national gastroenterology and hepatology cohort data^27^

		*N*	^ Valid %	NHWDS data (2016)^27^	Notes on comparability of the study cohort to NHWDS data
Profession	Gastroenterologist	70	75.3	730 (9.8%)	*Capturing ~10% of each cohort*
	In training	23	24.7	129 (17.8%)	
Age (years)	25–44	48	51.6	49 years average 17.5% >60 years	*Comparable to ~45 years average from respondent cohort.* *Similar representation of >60 age group*
	45–64	35	37.6		
	65+	10	10.8		
Sex	Male	62	66.7	79.3%	*↑ female representation, however based on data trends, increased % female workforce was anticipated*
	Female	30	32.3	20.7%	
	Other	1	1.1	—	
Years practicing in IBD speciality	0–4	34	36.6	—	
	5–9	13	14.0		
	10–15	12	12.9		
	15+	34	36.6		
Average hours in clinical practise/ week	0–9	2	2.2	43.7 h average	*Majority or respondent cohort (81%) aligns with NHWDS average (>30 h/week)*
	10–19	3	3.2		
	20–29	10	10.8		
	30+	75	80.6		
Where do you predominantly practise?	Public hospital	61	65.6	—	
	Private practice	32	34.4		
Geographical area serviced	Metropolitan	73	78.5	91.5% (MMM1)	Majority comparable areas. More *regional representation in respondent cohort (ie, 23% vs 5%)
	Regional	22	23.7	4.8% (MMM2)	
	Remote	2	2.2	3.7% (MMM3-7)	

*N* = 93 respondents in total.

IBD, inflammatory bowel disease; MMM, modified Monash model; NHWDS, National Health Workforce Dataset.

^Valid % refers to the percentage of the total number of responses for that survey item.

*NOTE: alignment on what is defined as regional/remote by MMM category is approximated.

### Current IBD Patient Satisfaction and Experiences with Alternative Therapies

Respondents asserted that their IBD patients had good overall satisfaction around the management of their condition (79% agreed patients satisfied; 12% neutral) with only a minority, suggesting that patients were unsatisfied (9%).

Respondents were largely supportive of use of complementary therapies (48% agreed; 27% neutral) and indicated that their patients reported various alternative and complementary therapies as being successful; primarily, restriction/exclusion dieting (77% agree successful), stress management (ie, CBT, mindfulness, and meditation; 72% agree) and pre- or probiotics (55% agree). A minority (27%) of the cohort endorsed cannabis as having been effective for their patients, with the remainder neutral (45%) or in disagreement (28%; Supplementary Figure 1).

### IBD Management with MC: Experiences and Attitudes

Over one third (39%) of respondents had experience with patients that currently use MC for their IBD management (Table 2). However, typically only a small number of experiences with MC patients were reported (mode 1–3 patients) with cannabis primarily being used as an adjunct to prescription pharmaceuticals (84%). Approximately half of the respondents reported having received MC enquiries from patients during the last 3 months (Table 2).

**Table 2.** Survey Respondent’s Experience with Medicinal Cannabis for IBD Management in Their Current Patient Cohorts

	Response	*N*	^Valid %
I have patients who currently use MC for IBD	Yes	36	39.1
	No	56	60.9
Number of patients using MC^#^	1–3 patients	18	66.7
	4–6 patients	6	22.2
	≥ 10 patients	3	11.1
How is MC being used by patients?	*Adjunct* to conventional treatments	32	84.2
	*In place of* conventional treatments	4	10.5
	Unsure	2	5.3
**Enquiries about MC in past 3 months**	None	33	41.8
(% of your total IBD patient cohort)	1%–9%	36	45.6
	10%–24%	10	12.6

MC, medicinal cannabis; IBD, inflammatory bowel disease.

^Valid % refers to the percentage of the total number of responses for that survey item.

^#^ *N* = 27 responses for this item only.

**Figure 1.**
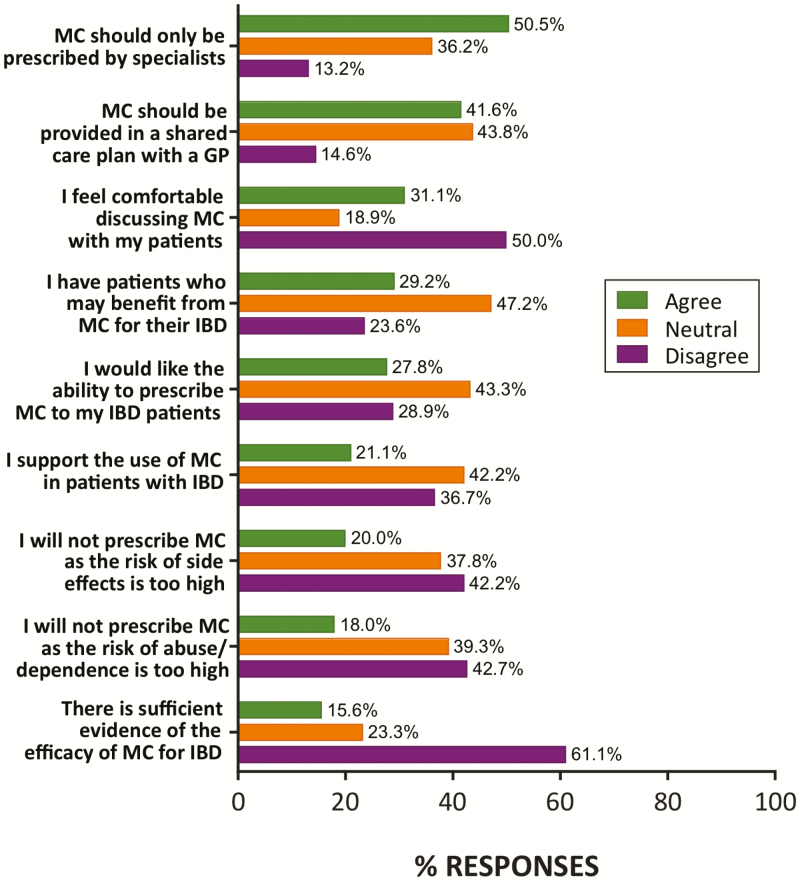
Attitudes and clinical experiences of IBD specialists with respect to MC for the management of IBD. While some specialists believe that there to be sufficient evidence for and patients who may benefit from the use of MC in IBD, support for use and willingness to prescribe were low. *N* = 89–91 responses per item. Percentage of total responses in each category (agree, neutral, and disagree) shown. MC, medicinal cannabis; IBD, inflammatory bowel disease; GP, general practitioner.

Overall support among specialists for the use of MC in the management of IBD was mixed. Respondents were largely neutral in their support of MC use in patients with IBD (42% neutral; 21% supportive), with only a minority wanting the ability to prescribe MC for their IBD patients (28%) (Fig. 1). This support was in line with 29% of respondents agreeing that they have IBD patients who may benefit from MC. Despite this level of support, only 16% thought there was sufficient current evidence for the efficacy of MC for IBD (Fig. 1).

Attitudes toward MC prescribing practice were mixed, with a slight majority believing that MC should only be prescribed by specialists (51%), and 42% supportive of a shared care plan involving a GP (44% neutral; Fig. 1).

Consistent with prior surveys, many respondents (50%) felt uncomfortable discussing MC with their patients (Fig. 1). Nonetheless, a majority (53%) said that they would encourage their IBD patients to take part in future clinical trials of MC, suggesting a willingness to investigate efficacy claims.

### Perceived Side Effects and Relative Hazards of MC

**Figure 2.**
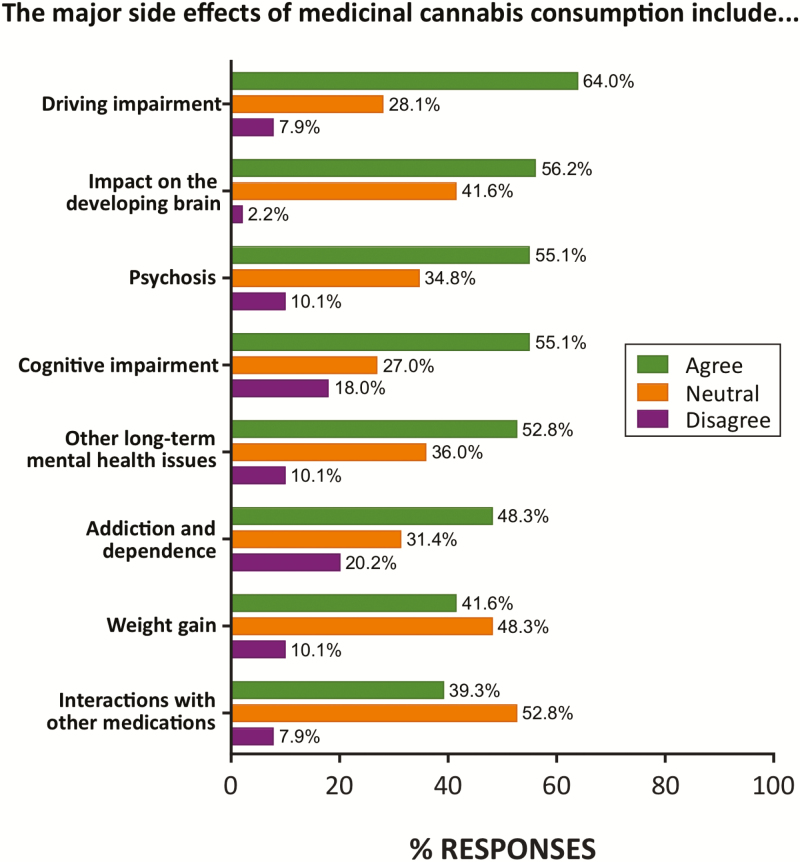
Perceived major side effects of MC in the survey cohort (*n* = 89). The most common perceived side effect was driving impairment, while close to half of respondents expressing concern over impact on developing brain, psychosis, cognitive impairment, and other long-term mental health issues. Percentage of total responses in each category is shown.

A majority of respondents agreed that the major side effects of MC consumption included impaired ability to drive (64%), impacts on the developing brain (56%), psychosis and cognitive impairment (55%) as well as addiction and dependence (48%; Fig. 2).

**Figure 3.**
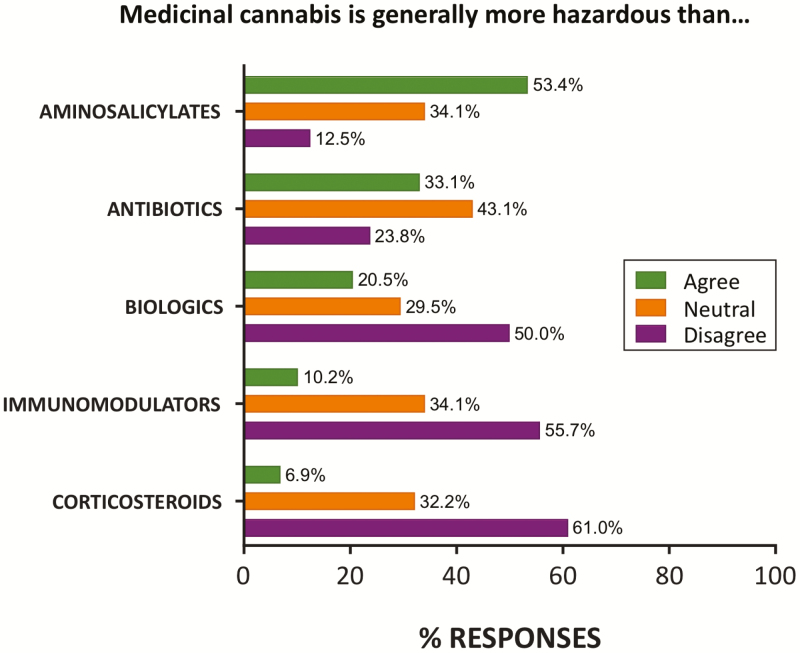
Perception of MC hazards compared to existing pharmaceutical classes used for IBD management. Over half the cohort rated MC as less hazardous than immunomodulators and corticosteroids. *N* = 88 responses (*n* = 87 for corticosteroids). Percentage of total responses in each category (agree, neutral, and disagree) shown.

Despite these perceived side effects, many did not see risk of abuse/dependence as a reason to avoid prescribing MC (43% disagreed), as with other side effects (42% disagreed; Fig 1). A surprisingly high proportion of respondents rated MC as less hazardous than existing prescription IBD treatments including corticosteroids (61%), immunomodulators (56%), and biologic therapies (50%). However, MC was rated more hazardous than aminosalicylates by 53% of respondents (Fig. 3).

### General Knowledge and Attitudes Toward MC and Regulation

**Figure 4.**
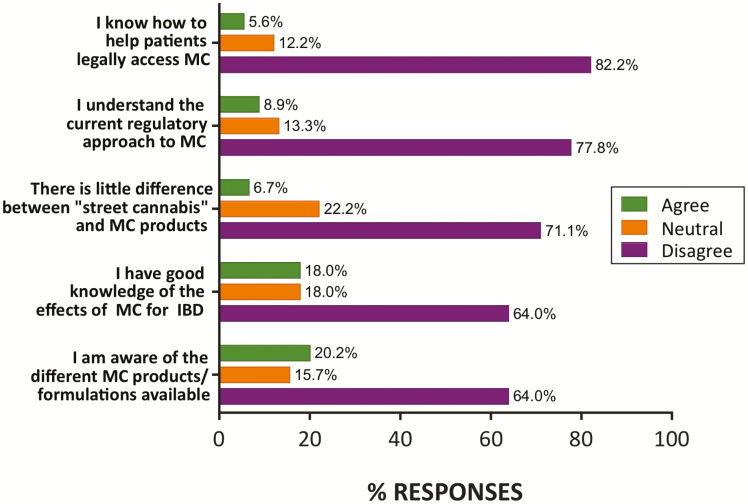
Respondent knowledge of MC and regulatory pathways in Australia. The majority of respondents were unaware of current regulatory approaches to MC and of product/formulations available. *N* = 90 responses (*n* = 89 for “formulation” and “knowledge of effects” items). Percentage of total responses shown. MC, medicinal cannabis; IBD, inflammatory bowel disease.

Respondents reported being largely unaware of the current regulatory approach to MC (71%) and about the different MC products/formulations available (64%). Only a minority (6%) had knowledge of how to access MC for their patients, and only 18% reported good knowledge of effects of MC in IBD (Fig. 4). Only a small minority of respondents (7%) agreed with the statement that there is little difference between “street cannabis” and MC products (Fig. 4).

### Open-Ended Comments

The 16 open-ended comments obtained included expression of skepticism around the level of evidence to support MC use (4/16), and the need for more education on this topic (4/16). Two comments were related to pediatric populations and concerns around children with IBD being included/excluded in future trials. Two comments stated that there is a role for MC in treating IBD. One response suggested that improvements in comorbid irritable bowel syndrome in their patients may explain improved symptom relief, rather than MC affecting IBD pathophysiology.

## Discussion

This is the first study to assess the knowledge and experiences of Australian gastroenterological specialists around the use of MC in IBD patients. This survey demonstrates that Australian gastroenterologists are mixed in their attitudes toward MC, with only a minority (28%) currently wishing to prescribe MC to their patients. This unwillingness to prescribe MC appears to be a multi-faceted phenomenon based on several factors: the lack of knowledge and familiarity of available products and access pathways; a disinclination to discuss MC with patients (neither of which are specific to the gastroenterology profession^26^); the equivocal evidence base supporting MC use; and significant concerns around side effects. We will now consider each of these factors in turn.

### Uncertainty Over MC Products and Access Pathways

Respondents had very limited awareness current regulatory approaches (7%), patient access pathways for legal products (6%), and the types of legally available MC products (20%; Fig. 4). This overall lack of knowledge clearly presents a barrier to prescribing MC products and discussing them in an informed way with patients. This perhaps reflects the recency of legislation supporting MC access in Australia and the fact that the therapeutic use of cannabis is a topic that is rarely included in formal medical specialist training.

The 6% of respondents expressing knowledge of access pathways is in line with the 10% awareness cited by Australian GPs in our 2018 survey.^26^ Problems around patient access pathways in Australia and associated lack of guidance for clinicians have been subject to recent criticism^28^ and there is clearly a need for more comprehensive education and guidance from government regulatory agencies and specialist colleges. Lack of knowledge of access pathways may also reflect the low motivation to prescribe: presumably if clinicians do not wish to prescribe MC then they will not take the time to discover how to do so.

There are currently more than 100 MC products available to prescribe in Australia through official access channels^7^ which makes selecting an appropriate product a considerable challenge without suitable education. It is not entirely clear how specialists can obtain quality information on how to effectively prescribe across such a wide diversity of products (eg, optimal cannabinoid profiles for use in IBD; different product formulations, strengths, and routes of administration; dose titration strategies; side effects) and this represents a significant concern around the effective use of these products.

### Difficulties Discussing MC with Patients

A substantial number of respondents (39%) reported having patients that currently use MC. Furthermore, more than half reported patients enquiring about MC availability for their IBD in the past 3 months. However, only a minority (31%) reported being comfortable discussing MC with their patients. This again reflects the need for education of specialists around MC so that they can communicate effectively and confidently with their patients who may well be already using MC products. Our survey of Australian GPs in 2018 showed a greater proportion of GPs felt comfortable discussing MC with their patients (49.1%)^26^ than the specialists in the current survey. Increased GP comfort may be a product of the variety of topics broached within the context of regular primary care compared with specialist care. Nonetheless, open communication is a pillar of patient care,^29^ and addressing the concerns of the patient is central to establishing an effective therapeutic relationship.

An additional factor to consider is the differences in perception of patient and clinician in terms of what constitutes an effective response to a new treatment, such as MC. Patients are of course focused on improved wellbeing which is often observed via change in symptoms, while clinicians are also monitoring endoscopic and biomarker changes, often imperceptible to the patient. This additional difference in priorities may make communication about alternative management strategies, such as MC, more challenging to discuss.

### Equivocal Evidence Supporting MC Use in IBD

Only a minority of specialists (16%) agreed that there was sufficient evidence for the efficacy of MC in IBD, and this provides another reason for the overall low support for MC use in IBD patients (21%) and low willingness to prescribe (28%). Randomized controlled trials to date suggest a variety of different MC products (inhaled cannabis,^20, 22^ CBD oil,^30^ and THC/CBD capsules^21^) can positively affect various co-morbid symptoms in IBD, but have not shown significant positive effects on disease markers and clinical remission. These trials, for the most part, represent low-quality evidence with small sample sizes, as shown in recent Cochrane reviews.^23, 24^ This situation will likely be clarified in the future as larger, more comprehensive trials are undertaken.

It is unclear the extent to which respondents are aware of the nuances of this existing evidence base but in any case their skepticism is consistent with the evolution of IBD therapy, aimed at halting disease progression rather than simple symptomatic improvement.^31^ This requires that novel therapeutics demonstrate an effect on biomarkers and/or endoscopic measures of inflammation rather than simply reducing symptoms such as cramps, pain, and insomnia.^32^ Such evidence, in relation to cannabinoids, only currently exists in preclinical research.^12–14^ Notably, in our parallel patient survey,^2^ MC appeared to be primarily improving symptoms associated with IBD (such as abdominal pain, anxiety, and sleep issues) (based on patient self-report) rather than directly affecting IBD pathophysiology and disease progression. These improvements in symptoms (sleep/pain/anxiety) as a result of MC use have also been reported in existing IBD RCTs^20, 21^ and other patient self-report observational studies.^16^ Additionally, clinical studies^33–35^ focused on the use of cannabinoid products specifically for pain management and anxiety have reported positive outcomes, further supporting the potential use of MC as a method of symptom control, outside of treatment of inflammation in IBD patients. Specialists were clearly interested in seeing the evidence base for MC in IBD grow with a majority (53%) supporting their patient involvement in relevant future clinical trials.

### Concern Regarding Side Effects of MC in IBD

Respondents generally agreed that MC had a range of side effects of potential concern including driving impairment,^36^ impacts on the developing brain, psychosis, and cognitive impairment^37^ (Fig. 2). Most of these are concerns are supported by available evidence, although concerns expressed around “weight gain” (42%) are largely unfounded given that cannabis users generally have a leaner phenotype than nonusers.^38, 39^

Risk of abuse/dependence, which 48% of respondents agreed was a major side effect of MC, was not, however, a significant deterrent to respondents prescribing MC (only 18% agreed that this would be a reason not to prescribe). In our previous survey of GPs, many of those who were unwilling to prescribe MC also agreed that risk of abuse/dependence was a concern.^26^ Cannabis dependence accounted for 4.6% of total drug-related hospitalizations in Australia between 2016 and 2017^40^ and is therefore a legitimate concern, although such hospitalizations may primarily reflect illicit recreational use rather than legitimate medicinal use of cannabis. Morbidity relating to cannabis dependence is lower than for prescription opioid and benzodiazepine dependence,^41^ as is mortality risk,^40^ and cannabis-related hospitalizations in Australia are lower than those attributable to benzodiazepines (7.6%) or opioids (6.3%).^40^ Such observations perhaps explain why risk of addiction is not seen as an insurmountable impediment to prescribing MC.

The side effect profile of many of the drugs used to conventionally manage IBD is significant. Long-term use of corticosteroids is associated with increased risk of serious infection, bone, and hepatic problems, while combined corticosteroid and immunomodulator use heightens risk of hospitalization and surgery relative to other IBD therapies (ie, aminosalicylates).^32^ Biologics are considered the most potent treatment for IBD, and thought to have a favorable risk–benefit ratio despite malignancies affecting some patients.^42^ Accordingly, many specialists saw MC as being less hazardous than corticosteroids (61%), immunomodulators (56%), and biologics (50%; Fig. 3) while 58% of respondents stated that the risk of side effects would not deter them from prescribing MC. Overall, specialists are clearly able to trade off risks for therapies that can halt disease progression.^43^

Concern about MC side effects by respondents may also reflect a general lack of knowledge around currently available MC products. These are often orally delivered oils (rather than smoked plant material), often with higher concentrations of the non-intoxicating cannabinoid CBD rather than the intoxicant THC. Not all specialists could clearly differentiate “street cannabis” from MC products and the side effects they endorsed are almost exclusively THC related, including driving impairment,^36^ impacts on the developing brain, psychosis, and cognitive impairment.^37^

In our recent parallel survey of Australian IBD patients using MC^2^, almost all patients (209/212) accessed MC through unregulated/illicit sources despite the availability of legal products through the TGA. Illicit products tend to be THC-dominant reflecting their sourcing from the recreational cannabis market.^44, 45^ Australian legal access pathways enable access to quality-controlled low-THC or THC-free products and allow the clinician to carefully manage cannabinoid content and dose (unlike in the United States where these decisions may be largely guided by the local dispensary). However, these official products are only being utilized by a small minority of IBD patients at present. This most likely reflects the dearth of specialists prepared to support an application for legal access, as well the high cost of the legal products in Australia.

### Future Directions

Over half of respondents would encourage their patients to be involved in clinical trials of MC products for IBD, and expressed interest in seeing the evidence base grow. Specialists are somewhat supportive of a shared care model with GPs, acknowledging the benefits of primary care and the topics which can be broached in this context. Patient demand for MC is set to increase, according to Australian medical authorities^46^ and so this is an issue that is only going to intensify. Clearly, it is necessary that education is made available to specialists around the evidence base in IBD, current access pathways, and available products. As clinical evidence grows, gastroenterologists may be more supportive of prescribed MC if it is provided with adequate TGA guidance^25, 26^ around the prescription and use of MC in IBD. The vast majority of MC users use MC concomitantly with their prescribed medication, indicating the importance of further research around polypharmacy and possible pharmacokinetic interactions between MC and IBD medications. It will also be important to distinguish between improvements of a symptomatic and pathophysiological nature, with patients clearly reporting a legitimate role for MC in the former context—a suggestion that is supported by commentary in the field^10^—while preclinical research identifies a role for novel cannabinoids, currently under development, in the latter.

### Limitations

Some limitations of our study should be noted. Firstly, we were unable to unambiguously verify the current registration status of respondents in the survey given the anonymity. In addition, we only sampled a small proportion of the Australian gastroenterological speciality (~10%) and can only comment on our sample and infer this is representative of the larger Australian cohort, which does not account for potential nonresponse bias in our study.

## CONCLUSIONS

We have produced a snapshot of the attitudes and knowledge of Australian IBD specialists toward MC use in IBD management that suggests current caution and some discomfort, likely due to lack of familiarity and education around use of MC products and their access routes, the absence of compelling evidence for MC efficacy, and potential side effects. However, this cohort of specialists is clearly interested in further clinical investigation of MC and is open to education about this topic. It is anticipated that the specialist–patient interface around MC is an area where rapid changes will be observed within a relatively short timeframe.

## Supplementary Material

otaa045_suppl_Supplementary_Material

## Data Availability

Anonymized survey data are available at the Open Science Framework data repository; project ID: Attitudes and Knowledge of Australian Gastroenterologists around use of Medicinal Cannabis for Inflammatory Bowel Disease at https://osf.io/3xfuz/?view_only=c4597b1ad28e4f46a0387291c0c0ece0
