# Supplementary material for: Attitudes and Knowledge of Australian Gastroenterologists Around the Use of Medicinal Cannabis for Inflammatory Bowel Disease
Source: Crohns Colitis 360. 2020 Jun 8;2(2):otaa045. doi: 10.1093/crocol/otaa045 (PMC9802365; doi:10.1093/crocol/otaa045)
Supplement: otaa045_suppl_Supplementary_Material [file otaa045_suppl_supplementary_material.pdf]

LAMBERT INITIATIVE
FOR CANNABINOID THERAPEUTICS

THE UNIVERSITY OF SYDNEY

MEDICINAL CANNABIS SURVEY FOR IBD SPECIALISTS

On behalf of the University of Sydney's Lambert Initiative for Cannabinoid Therapeutics, you are invited to take part in a research study investigating gastrointestinal specialist experiences and perceptions of medicinal cannabis for Inflammatory Bowel Disease. The results of this study may be used to inform policy decisions and develop educational material to meet the specific needs of specialists, as well as to inform interest in participating in our clinical research.

Participation in this study is completely voluntary and involves completion of a short anonymous survey. If you would like to participate, simply complete the paper questionnaire and mail back using the provided reply paid envelope. Submitting your completed questionnaire is an indication of your consent to participate in the study.

Alternatively, you may opt to complete the survey online through a University of Sydney hosted survey site. Go to: ***redcap.sydney.edu.au/surveys/?s=EXK93AMXMM***

All survey responses are anonymous when submitted through either format (paper or online).
*Please select **one** answer per question.*

I confirm I have not and will not complete this survey online through the link provided. I will complete only this paper survey: ☐ No ☐ Yes

ABOUT YOU

**Profession**	Gastroenterologist ☐	Colorectal surgeon ☐	In training ☐	Other ☐
**Age category**	18-24 ☐	25-44 ☐	45-64 ☐	65+ ☐
**Sex**	M ☐	F ☐	Other ☐	
**Years in gastrointestinal specialty**	0-4 ☐	5-9 ☐	10-15 ☐	15+ ☐
**Average hours spend in clinical practice per week**	0-9 ☐	10-19 ☐	20-29 ☐	30+ ☐
**Where do you predominantly practice?**	Public hospital ☐	Private practice ☐		
**Geographical area serviced**	Metro ☐	Regional ☐	Remote ☐	

YOUR CURRENT IBD PATIENT COHORT

	Strongly disagree	Slightly disagree	Neutral	Slightly agree	Strongly agree
1. My patients are satisfied with their current IBD drug management regimen	☐	☐	☐	☐	☐
2. I support my patients using alternative/complementary therapies or supplements to manage their IBD	☐	☐	☐	☐	☐
3. My patients have reported success in managing their IBD symptoms with the following alternative/complementary therapies:					
Prebiotics or probiotics	☐	☐	☐	☐	☐
Fish oil (omega-3 fatty acid) supplement	☐	☐	☐	☐	☐
Herbal or dietary supplements	☐	☐	☐	☐	☐
Restriction/exclusion diet (e.g. low FODMAP diet)	☐	☐	☐	☐	☐
Stress management (e.g. cognitive behavioural therapy, mindfulness meditation)	☐	☐	☐	☐	☐
Feacal microbiota transplantation (FMT)	☐	☐	☐	☐	☐
Traditional Chinese medicine/acupuncture or moxibustion	☐	☐	☐	☐	☐
Chiropractic/osteopathy/massage	☐	☐	☐	☐	☐
Cannabis	☐	☐	☐	☐	☐
Gut-directed hypnotherapy	☐	☐	☐	☐	☐
None of the above	☐	☐	☐	☐	☐

Other (describe): ____________________

4. I have patients who currently use cannabis for their IBD — No ☐ Yes ☐ — If yes, how many patients? ____________

THE UNIVERSITY OF SYDNEY

IF answered YES to Q4 please complete Q4b and Q4c. IF answered NO to Q4 please proceed to Q5 below:

4b . Of your patients using cannabis, how is it most commonly being used to treat their symptoms?

In combination with conventional treatments (adjunct)	☐
In place of all conventional treament (stand-alone)	☐
Unsure	☐

4c . Patients using cannabis for their IBD have reported what/if any change to the following symptoms:

	Significant Worsening	Slight Worsening	No change	Slight Improvement	Significant Improvement	Unknown	N/A
Anxiety	☐	☐	☐	☐	☐	☐	☐
Depression	☐	☐	☐	☐	☐	☐	☐
Memory	☐	☐	☐	☐	☐	☐	☐
Fatigue	☐	☐	☐	☐	☐	☐	☐
Appetite	☐	☐	☐	☐	☐	☐	☐
Nausea/vomiting	☐	☐	☐	☐	☐	☐	☐
Abnominal Pain	☐	☐	☐	☐	☐	☐	☐
Bloating	☐	☐	☐	☐	☐	☐	☐
Cramping	☐	☐	☐	☐	☐	☐	☐
Stool consistency	☐	☐	☐	☐	☐	☐	☐
Stool frequency	☐	☐	☐	☐	☐	☐	☐
Urgency	☐	☐	☐	☐	☐	☐	☐
Obstructive symptoms (fistula, abscess, stricture)	☐	☐	☐	☐	☐	☐	☐
Rectal bleeding	☐	☐	☐	☐	☐	☐	☐
Weight loss	☐	☐	☐	☐	☐	☐	☐
Sleep issues	☐	☐	☐	☐	☐	☐	☐
Relapse potential	☐	☐	☐	☐	☐	☐	☐
Quality of Life	☐	☐	☐	☐	☐	☐	☐
Other: ________							

	None	1-9%	10-24%	25-49%	50-75%	> 75%
**5 . In the past 3 months, how many of your patients have enquired about using medicinal cannabis to treat their IBD symptoms?**	☐	☐	☐	☐	☐	☐

YOUR PERSPECTIVES ON MEDICINAL CANNABIS

	Strongly disagree	Slightly disagree	Neutral	Slightly agree	Strongly agree
**6 . I have patients who may benefit from medicinal cannabis for their IBD**	☐	☐	☐	☐	☐
**7 . I feel comfortable discussing medicinal cannabis with my patients**	☐	☐	☐	☐	☐
**8 . I have good knowledge around the effects of medicinal cannabis for IBD**	☐	☐	☐	☐	☐
**9 . I support the use of medicinal cannabis in patients with IBD**	☐	☐	☐	☐	☐
**10 . I am aware of the different medicinal cannabis products and formulations currently available**	☐	☐	☐	☐	☐
**11 . I would like the ability to prescribe medicinal cannabis products to my IBD patients**	☐	☐	☐	☐	☐
**12 . Medicinal cannabis should only be prescribed by specialists**	☐	☐	☐	☐	☐
**13 . Medicinal cannabis should be provided in "shared care" with a GP**	☐	☐	☐	☐	☐
**14 . I know how to help patients legally access medicinal cannabis**	☐	☐	☐	☐	☐
**15 . I understand the current regulatory approach to medicinal cannabis**	☐	☐	☐	☐	☐
**16 . There is little difference between "street cannabis" and medicinal cannabis products**	☐	☐	☐	☐	☐
**17 . I will not prescribe medicinal cannabis as the risk of abuse and dependence is too high**	☐	☐	☐	☐	☐
**18 . I will not prescribe medicinal cannabis as the risk of side effects (other than abuse and dependence) is too high**	☐	☐	☐	☐	☐
**19 . There is sufficient scientific evidence of the efficacy of medicinal cannabis for IBD**	☐	☐	☐	☐	☐

20 . The major side effects of medicinal cannabis consumption include:	Strongly disagree	Slightly disagree	Neutral	Slightly agree	Strongly agree
Addiction and dependence	☐	☐	☐	☐	☐
Cognitive impairment	☐	☐	☐	☐	☐
Driving impairment	☐	☐	☐	☐	☐
Weight gain	☐	☐	☐	☐	☐
Psychosis	☐	☐	☐	☐	☐
Other long-term mental health issues	☐	☐	☐	☐	☐
Interactions with other medications	☐	☐	☐	☐	☐
Impact on the developing brain	☐	☐	☐	☐	☐

20 . Medicinal cannabis is generally more hazardous than:	Strongly disagree	Slightly disagree	Neutral	Slightly agree	Strongly agree
Aminosalicylates	☐	☐	☐	☐	☐
Antibiotics	☐	☐	☐	☐	☐
Biologic Therapies (e.g. Antibody-based treatments)	☐	☐	☐	☐	☐
Corticosteroids	☐	☐	☐	☐	☐
Immunomodulators	☐	☐	☐	☐	☐

21 . Would you be interested in encouraging your IBD patients to take part in future clinical trials to test the effects of medicinal cannabis as a treatment for IBD?

If you answer YES please provide your email address below on the lower portion of this page. This will be removed from your survey responses by a member of the study team prior to the survey being recorded or analysed to ensure data anonymity.

No ☐ Yes ☐

OPEN ENDED COMMENTS

Please use this space to offer any comments, opinions or observations about medicinal cannabis in Australia

Study Team to detach here

THIS SECTION OF SURVEY TO BE REMOVED PRIOR TO DATA RECORDING OF THIS SURVEY TO ENSURE ANONYMITY

IF you answered YES to Q21; "Would you be interested in encouraging your IBD patients to take part in future clinical trials" please provide your contact email address below:

Email address: ____________________
